# Appetitive reversal learning differences of two honey bee subspecies with different foraging behaviors

**DOI:** 10.7717/peerj.5918

**Published:** 2018-11-21

**Authors:** Eddie Pérez Claudio, Yoselyn Rodriguez-Cruz, Okan Can Arslan, Tugrul Giray, José Luis Agosto Rivera, Meral Kence, Harrington Wells, Charles I. Abramson

**Affiliations:** 1 Department of Biology, Universidad de Puerto Rico, Recinto de Rio Piedras, San Juan, PR, USA; 2 Department of Science and Mathematics, Universidad Interamericana de Puerto Rico, Bayamon, PR, USA; 3 Department of Biology, Middle East Technical University, Ankara, Turkey; 4 Department of Biology, University of Puerto Rico, San Juan, PR, USA; 5 Department of Biological Science, University of Tulsa, Tulsa, OK, USA; 6 Department of Psychology, Oklahoma State University, Stillwater, OK, USA

**Keywords:** Associative learning, Behavioral ecology, Social insect, Hymenoptera, Learning plasticity, Specialist, Extinction, Memory, Generalist

## Abstract

We aimed to examine mechanistically the observed foraging differences across two honey bee, *Apis mellifera*, subspecies using the proboscis extension response assay. Specifically, we compared differences in appetitive reversal learning ability between honey bee subspecies: *Apis mellifera caucasica* (Pollman), and *Apis mellifera syriaca* (Skorikov) in a “common garden” apiary. It was hypothesized that specific learning differences could explain previously observed foraging behavior differences of these subspecies: *A.m. caucasica* switches between different flower color morphs in response to reward variability, and *A.m. syriaca* does not switch. We suggest that flower constancy allows reduced exposure by minimizing search and handling time, whereas plasticity is important when maximizing harvest in preparation for long winter is at a premium. In the initial or *Acquisition* phase of the test we examined specifically discrimination learning, where bees were trained to respond to a paired conditioned stimulus with an unconditioned stimulus and not to respond to a second conditioned stimulus that is not followed by an unconditioned stimulus. We found no significant differences among the subspecies in the *Acquisition* phase in appetitive learning. During the second, *Reversal* phase of the experiment, where flexibility in association was tested, the paired and unpaired conditioned stimuli were reversed. During the *Reversal* phase *A.m. syriaca* showed a reduced ability to learn the reverse association in the appetitive learning task. This observation is consistent with the hypothesis that *A.m. syriaca* foragers cannot change the foraging choice because of lack of flexibility in appetitive associations under changing contingencies. Interestingly, both subspecies continued responding to the previously rewarded conditioned stimulus in the reversal phase. We discuss potential ecological correlates and molecular underpinnings of these differences in learning across the two subspecies. In addition, in a supplemental experiment we demonstrated that these differences in appetitive reversal learning do not occur in other learning contexts.

## Introduction

A honey bee colony shifts its foraging effort as the floral resources come and go in the environment (see Seeley, 1995). This dynamic allocation of foragers is thought to be adaptive since resources are harvested maximally. The basis of this constant response to changes in floral resources is the preference and foraging decisions of individual honey bees. Several mechanisms involving learning have been shown to be important in decisions of individual foragers (Ferguson, Cobey & Smith, 2001). We examined whether plasticity in appetitive learning will differentiate bees of *Apis mellifera caucasica* subspecies that switch foraging preferences with ease from bees of *Apis mellifera syriaca* subspecies that do not switch even when reward contingencies change (see Çakmak et al., 2010).

Both specialist strategy of *A.m. syriaca*, and generalist strategy of *A.m. caucasica* could be adaptive in their respective environments. The hypothesis is that specializing on a single flower type makes the bee faster both in finding the flower and in handling the flower, and thus decreases the time spent outside, at risk, or exposure to predators. Therefore, appetitive learning flexibility in the specialist subspecies, *A.m. syriaca* should be reduced to keep the bee focused on a single flower type. Alternately, in a low risk environment, a fully plastic foraging choice toward the most rewarding resources is the best solution, and favors greater learning plasticity in the generalist subspecies, *A.m. caucasica*. Then predation risk sets limits to plasticity in foraging choice (DeWitt, Sih & Wilson, 1998; Murren et al., 2015).

Honey bees live in a wide range of habitats, extending from tropical to subarctic, either because of human intervention or because of evolutionary history of the populations (Whitfield et al., 2006; Wallberg et al., 2014). These genetically distinct populations are recognized as subspecies or races. Bringing members of different subspecies together for experiments revealed many genetic differences in behavior and its regulation (Giray et al., 2000; Brillet et al., 2002; Alaux et al., 2009; Çakmak et al., 2009, 2010; Kence et al., 2013; Büchler et al., 2014). Foraging choice differences across two subspecies from Turkey provides the ideal situation to test the underlying learning plasticity differences across specialists and generalists. Previously, *A.m. syriaca* and *A.m. caucasica* bees have been studied for genetic, colony and behavioral differences (genetics: Bodur, Kence & Kence, 2007; foraging behavior: Çakmak et al., 2009; colony traits: Çakmak et al., 2010; Kence et al., 2013).

The bees from the subspecies *A.m. syriaca* inhabit southeast Anatolia, a generally dry habitat with longer seasonal foraging periods constrained by periodic blooms of one or few flowers (Kandemir, Kence & Kence, 2000; Kandemir et al., 2006). For foraging *A.m. syriaca* bees, minimizing predation risk is important. In this region, there is a predatory wasp that can capture foraging honey bees, and bees of this region are demonstrated to have specific behavioral adaptations against this Vespa species, such as reducing foraging activity (Ishay, Bytinski-Salz & Shulov, 1967; Butler, 1974; Ruttner, 1988; Roubik, 1992; Çakmak, Wells & Firatli, 1998). This response is absent in *A.m. mellifera* (Matsuura & Sakagami, 1973). In contrast, the bees from the subspecies *A.m. caucasica* inhabit temperate deciduous forests in the northeast of Anatolia and the eastern Black Sea coast regions of Turkey. Weather in these regions limits foraging to a short, 3-month seasonal period, making it important to maximize collection rate.

One specific type of plasticity in learning, reversal learning, has been examined because of its potential relevance to tracking changing foraging resources (Ferguson, Cobey & Smith, 2001). The bees learn to associate a stimulus (a floral odor) with a reward and learn to discriminate this from a second odor not associated with reward. Later bees are asked to switch the odor associations. Reversal learning measures behavioral flexibility, and either single or multiple reversions, and either two or more choices are utilized to examine the extent of flexibility (Izquierdo et al., 2017). In comparison of bees of different ages (Ben-Shahar et al., 2000), selected lines (Ferguson, Cobey & Smith, 2001), and subspecies (Abramson et al., 2015), rate of reversal appears to differ, albeit the shape of reversal appears to remain similar (see Fig. S1).

**Figure 1:**
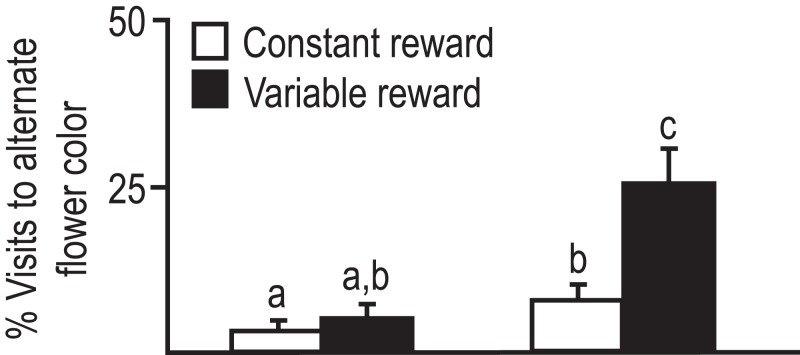
Foraging visits of bees from two subspecies to alternate flowers when preferred flower provides constant or variable amounts of nectar reward. Average percent visits to alternate flower color was significantly less for *A.m. syriaca* than *caucasica*. Bees first visited blue, white or yellow flowers. Later they visited alternates or initial preferred flowers with either constant reward (two μl 1M sucrose) or variable reward (only one of three flowers with six μl reward). Sample size: six colonies/subspecies, 30–35 bees/colony, 30–40 choices/bee. Error bars = SE. Factorial ANOVA indicated significant subspecies differences. Groups with different letters above bars are different at *p* < 0.05 (Çakmak et al., 2010).

In the context of foraging behavior, reversal learning is similar to when a bee visits one flower providing nectar at that time, and later in the day switch to a different flower, that is, providing nectar then (Wagner et al., 2013). In addition, the response of bees to variability in nectar availability is similar to the response of other organisms such as vertebrates to variable reward or resources under experimental or natural conditions (Commons, Kacelnik & Shettleworth, 1987). For instance, if the constant forage rate would provide energetic needs, organisms are likely to abandon variable reward for constant reward (Caraco, 1981; Zalocusky et al., 2016). In previous work, we have demonstrated that bees from the temperate subspecies *A.m. caucasica* a more likely to switch to a different flower color morph. In contrast, bees from the subtropical subspecies *A.m. syriaca* are not sensitive to variability in reward, and continue to visit the same flower morph even when rate of reward is one in three visits (Çakmak et al., 2010; Fig. 1).

We hypothesized that flower constancy even when faced with variable reward could be due to learning and memory differences of *A.m. syriaca* bees from other bees, including *A.m. caucasica*. We used the proboscis extension response (PER) conditioning (Abramson et al., 2015) assay to examine differences in appetitive learning behavior across bees from colonies of both subspecies maintained in a “common garden” apiary (Kence et al., 2013).

## Materials and Methods

### Experimental design

Proboscis extension response conditioning experiments were performed between June and July 2014 at the Middle East Technical University in Ankara, Turkey. In a preliminary work, we also examined reversal in a non-appetitive aversive learning test, electric shock avoidance conditioning (ESA, Agarwal et al., 2011; Dinges et al., 2013). To control for calendar variables associated with weather and field conditions, both PER and ESA (Fig. S2; Supplemental Results) conditioning assays were run simultaneously. In the ESA series, we investigated the reversal of spatial avoidance learning in honey bees confined to a shuttle box.

Foragers of two subspecies populations in Turkey were used. One subspecies was *A.m. caucasica*, and the other subspecies was *A.m. syriaca*. Both subspecies were maintained in a common garden under similar environmental conditions. Great care is taken to ensure that the subspecies lines are maintained and this is confirmed by use of genetic and morphological measurements, and acquiring new colonies or naturally mated queens from the geographically separated (>600 miles) locations (Kence et al., 2013). We used three colonies from each honey bee subspecies to increase genetic variation within the samples for a total of 261 individuals that were tested in learning and memory assays. A total of 137 bees, divided in two equal groups (but for one bee), one for each subspecies, were recruited for the PER assays where each experimental group consisted of 12 individuals, except in occasion one or two bees were eliminated when not responsive. A total of 124 bees, divided in four equal groups, two for each subspecies, were recruited for the supplemental ESA assays (Fig. S2; Supplemental Results) where each experimental group consisted of up to 34 individuals.

### Proboscis extension response reversal learning

In these experiments there are two phases, acquisition and reversal. In the acquisition phase, we examined differential conditioning, where we trained the honey bees to discriminate between two conditioned stimuli (CS)—one paired with a sucrose feeding (CS+), and the other not (CS−). Following this, in the reversal phase, we reversed the CS+ and CS− roles such that the CS+ is now the CS− and the CS− is now the CS+.

One CS consisted of lavender odor (Gilbertie’s, Southampton, NY, USA) and the other cinnamon odor (Gilbertie’s, Southampton, NY, USA). The rationale behind the use of these odors is that we have found them effective in our previous discrimination experiments in Turkey (Abramson et al., 2008, 2010, 2015). The CS odor was applied to a 1 cm^2^ piece of Whatman (#4) filter paper using a wooden dowel and then secured to the plunger of a 20 cc plastic syringe with an uncoated metal thumbtack. Our earlier work demonstrated this procedure produces reliable results consistent with automated methods (Abramson & Boyd, 2001).

To remain consistent with our previous work: (1) a non-overlap procedure was used in which the CS terminated before the US (Abramson et al., 1997), (2) the CS duration was 3 s and the US duration was 2 s, and (3) the intertrial interval between CS presentations was a fixed 5-min interval. During the initial discrimination learning phase, each bee received six trials each with lavender and cinnamon for a total of 12 trials. During the reversal phase in which the role of the CSs were reversed, bees received six trials each with lavender and cinnamon for an additional 12 trials. The order of CS+ and CS− presentations were pseudorandom and identical for each bee. We used the order: initial discrimination training: CS+, CS−, CS−, CS+, CS−, CS+, CS+, CS−, CS+, CS−, CS−, CS+, reversal training: CS−, CS+, CS+, CS−, CS+, CS−, CS−, CS+, CS−, CS+, CS+, CS− for a total of 24 trials (12 CS+ and 12 CS−).

Honey bees from both subspecies were captured one day before the experiment. They were captured in glass vials and placed in ice. While sedated they were harnessed in metal tubes with a piece of duct tape placed between the head and thorax. Once awake, they were fed 1.5M sucrose solution in water until satiated and set aside in a fume hood. On the day of the experiment, the bees were removed from the fume hood and were placed in batches consisting of about 12 bees.

A conditioning trial was initiated by picking up a bee from its position in the batch and placing it in the fume hood. The purpose of the fume hood was to eliminate any lingering CS odors. After a few seconds, but never immediately upon placement, the CS was administered for 3 s and was immediately followed by the US. This procedure was necessary as bees can associate the “placement” with a feeding. The US was presented by touching the bee’s antennae with a filter paper strip containing 1.5M sucrose and bees were allowed to lick the filter paper for 2 s after extending their proboscis. At the end of the 2-s feeding, the bee was removed from fume hood and returned to its place in the batch at which time the next bee in the batch was placed in fume hood for its trial. This process continued until all the subjects in the batch received the required number of conditioning trials. During each trial, responses to the CS were recorded visually. If the bee extended its proboscis during the CS presentation, a positive response was recorded. If the bee did not extend its proboscis during the CS presentation, a “0” response was recorded. The experiment was run blind as the experimenter did not know what subspecies was being trained. This was assured by using a code for source colony, and by using help of individuals who would not run the experiment in fixing bees into holders in preparation for PER conditioning.

Each experiment consisted of two phases. The stage where memory of the paradigm was being acquired for the first time was termed *Acquisition* phase. The step where we reverse the paradigm was termed *Reversal* Phase. During each trial we presented a CS+ and a CS−, each CS was a different odor. We used a model with two sets of experiments where each odor had the role of acquisition phase CS+ (Initial CS+) or acquisition phase CS− (Initial CS−) thus creating a counterbalance. The measured value was the PER response.

### Supplemental electric shock avoidance assay

This experiment had two phases of 5 min each for a total of 10 min. During acquisition phase, individuals were presented two colors, one as the punishment conditioned stimulus (CS+), this color was paired with electric shock (unconditioned stimuli), and the other as the no punishment conditioned stimulus (CS−), this color was not paired with electric shock. Here individuals learn to avoid punishment or one of the colors. That is to say, the bee learns to stay on one side of the box and not on the other. During the second or Reversal phase, the colors for the CS+ and CS− were switched. Now the acquisition phase CS+ is the reversal phase CS− and the acquisition phase CS− is the reversal phase CS+. We do the switch by changing the side/color of the box that receives shock, and not by moving the colors, this way we avoid confounding position and color effects. Moreover, by moving the shock from one side of the box to another, the bee can only avoid the shock by making an active response; by moving from one side to the other. We omitted the test phase (period of time without shock) that is usually performed after a trial or phase that demonstrates memory (Agarwal et al., 2011; Dinges et al., 2013; Giannoni-Guzmán et al., 2014). This was done to prevent the memory extinction process from interfering with the reversal phase.

To analyze the results from these experiments we first confirmed there is no color preference by bees from either subspecies when either blue or yellow was the CS− during acquisition and reversal phases. Because we did not observe significant differences (see results in the Fig. S2; Supplemental Results), color was not included as a variable in subsequent analyses. Instead, the first color associated with punishment is A+, and the second or reversal phase this is A−, whereas the alternate color becomes B+.

We used a shuttle box apparatus as described before (Agarwal et al., 2011; Giannoni-Guzmán et al., 2014). The shuttle box measured 15 cm long by two cm wide and contained an electric shock grid with wires spaced 0.35 cm apart. The shock was presented to only one side of the apparatus identified by a specific color. Shock intensity was 6 V 50 mA DC from an analog power supply and was low enough not to produce a sting reflex. In one half of the shuttle box a color (CS) is paired with electric shock (US) to create a CS+, on the other half another color (CS) is not paired with the electric shock (US) to create a CS−. Time spent on the shock side was recorded by an observer, one observer for each individual. We used blue and yellow as we know from our previous experiments that bees can readily distinguish between them. We measured the mean amount of time spent on the shock side in sets of 60 s for a total of five sets or 300 s as was done previously (Agarwal et al., 2011).

### Statistical analysis

Statistical analyses were performed using the GraphPad Prism 6 statistical software program. Analyses of the data from PER and the ESA assays were done with: two-way repeated measures ANOVA, Wilcoxon- matched-pairs signed rank test, and Student’s *T*-test. We tested the data for significant phase (Acquisiton vs Reversal), subspecies, and interaction effects. In the case of ANOVA, a post-hoc Tukey–HSD test was used to examine trial to trial differences. We verified fit to a normal distribution using the Shapiro–Wilk’s W test.

## Results

Two-way ANOVA comparison shows *A.m. caucasica* has no significant odor preference between lavender and cinnamon for the Initial CS+ (*F*_(1,54)_ = 0.6779, *ω*^2^ = 0.2454, *p* = 0.4139; *N*_1(Lavender)_ = 27, *N*_2(Cinnamon)_ = 29) or the Initial CS− (*F*_(1,54)_ = 0.04922, *ω*^2^ = 0.01582, *p* = 0.8253; *N*_1(Cinnamon)_ = 27, *N*_2(Lavender)_ = 29). Likewise, *A.m. syriaca* showed no significant odor preference between lavender and cinnamon for the Initial CS+ (*F*_(1,54)_ = 0.2687, *ω*^2^ = 0.0628, *p* = 0.6063; *N*_1(Lavender)_ = 27, *N*_2(Cinnamon)_ = 29) or the Initial CS− (*F*_(1,54)_ = 1.626, *ω*^2^ = 0.6175, *p* = 0.2077; *N*_1(Cinnamon)_ = 27, *N*_2(Lavender)_ = 29). As a result, type of odor was excluded from further consideration, and the first CS+ odor is simply coded as A+, and the second CS+ as B+, the odors that are CS− are then B− in the acquisition phase, and A− in the reversal phase.

**Figure 2:**
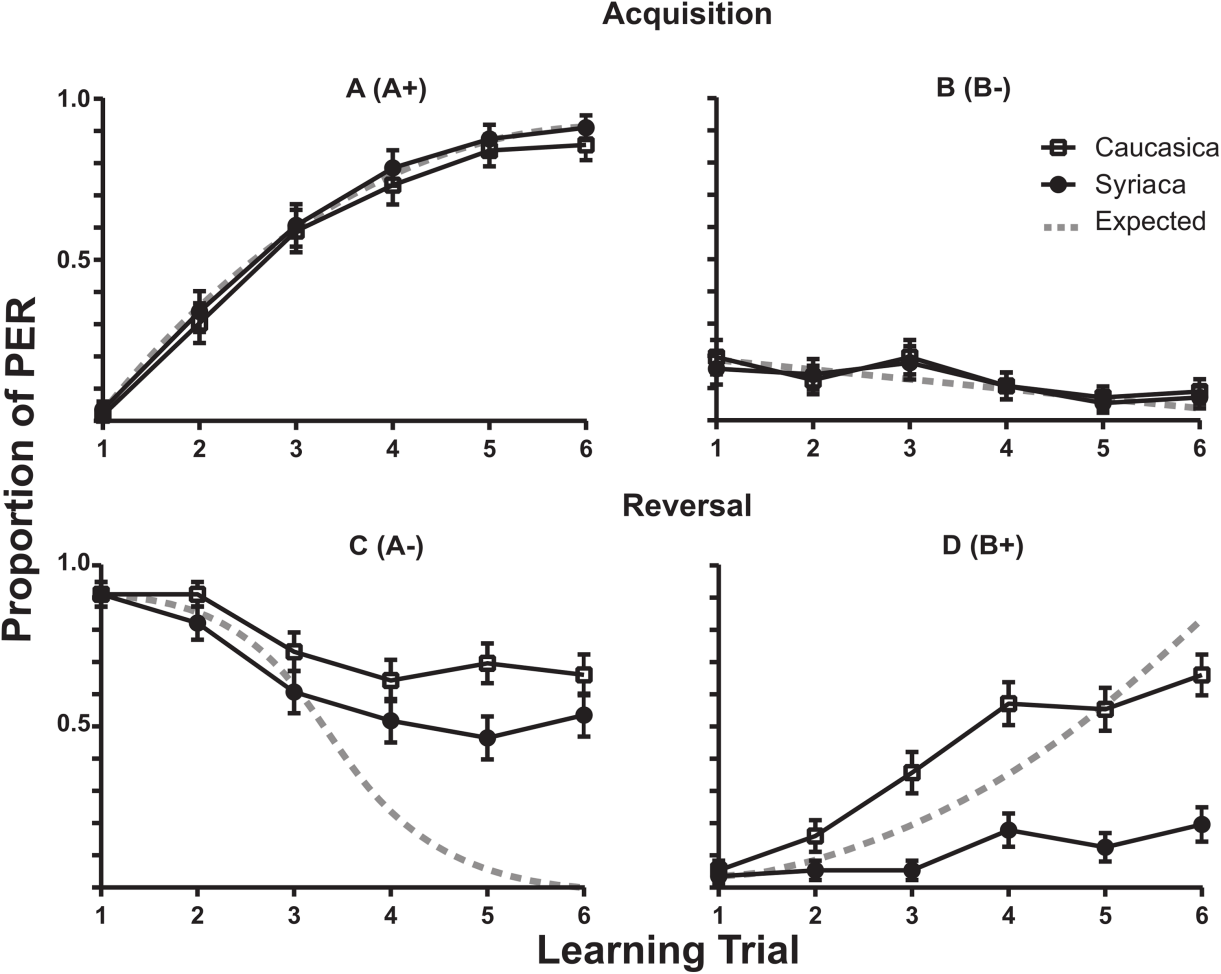
Proboscis Extension Response of *A.m. caucasica* and *A.m syriaca* during a reversal learning test. Comparison of responses to odors A and B between honey bee subspecies *A.m. caucasica* and *A.m. syriaca* during a proboscis extension response (PER) assay. Each data point shows the percentage (± standard error) of bees that showed PER during the assay. A (A+) and B (B−) show acquisition phase, and C (A−) and D (B+) show reversal phase. During the Reversal for A (C), ANOVA test shows differences at the subspecies level in the extinction rate (*p*-value = 0.0310, *F*(1,110) = 4.777). During the Reversal for B (D), ANOVA test shows differences in the learning rate at the subspecies level (*p*-value < 0.0001, *F*(1,110) = 44.43).

The learning rates for the A+ in the Acquisition phase for both subspecies members are described in Fig. 2A (A+). The fewer response of proboscis extension by members of both subspecies to B− in the acquisition phase is plotted in Fig. 2B (B−). The reversal phase responses are shown in Fig. 2C (B+). The reversal phase extinction of odor A (A−) showed that after six trials of where no reward was presented following odor A (A−), bees of both subspecies continued to present PER response above 50% of the trials (Fig. 2D (A−)). During this phase *A.m. syriaca* reached significantly lower response rates in comparison to *A.m. caucasica* (*F*_(1,110)_ = 4.777, *ω*^2^ = 1.607, *p* = 0.0310; *N*_1(Caucasica)_ = 56, *N*_2(Syriaca)_ = 56).

## Discussion

The most significant finding of this study is that appetitive olfactory reversal learning differences across honey bee subspecies match differences in their foraging plasticity. In appetitive olfactory reversal learning, bees from the subtropical subspecies *A.m. syriaca* do not show reversal, specifically they do not form association for the odor that is rewarded in the reversal phase. Unlike the typical reversal response of other organisms, such as other bee subspecies (see below), bees in this study continued to respond to the previously rewarded but now unrewarded odor in the reversal phase. Should these responses occur in the context of foraging, *A.m. syriaca* bees are expected to visit only flowers similar to a first learned flower. *A.m. caucasica* bees would be expected to visit an expanding repertoire of flowers with different features. These results suggest molecular substrates of learning and memory to be candidates for selection in adaptation to specific ecological conditions.

### Specific learning differences across populations

This study is, to our knowledge, the first to demonstrate specific learning plasticity differences across genetically distinct populations of the same species. This could be due both to comparison of populations from contrasting environmental conditions and to use of a complex learning paradigm. We found that bees from both subspecies has a similar learning rate for the A+ in the Acquisition phase (see Fig. 2A (A+)). We also found that both subspecies showed discrimination and did not respond by proboscis extension to B− in the acquisition phase (see Fig. 2B (B−)). Surprisingly we found that during Reversal Phase *A.m. syriaca*’s acquisition of B+ is impaired (Fig. 2D (B+)). This is unique to *A.m. syriaca* as can be seen when our results are compared with those of similar experiments in the European honey bee from North America (a mix of the European *A. mellifera* subspecies, Ben-Shahar et al., 2000; Fig. S1) or *A.m. anatoliaca* (Abramson et al., 2015). In contrast, in this study especially the Reversal Phase extinction of odor A (A−) was different, in that complete extinction did not occur, and extinction was slower for both *A.m. caucasica* and *A.m. syriaca* in comparison to bees from other subspecies (Fig. S1, also see Fig. 2C (A−)). Yet another difference was for *A. syriaca* in the reversal phase conditioning of odor B (B+), where *A.m. caucasica* showed the typical learning curve and responded with PER to B+, the *A.m. syriaca* continued withholding PER (Fig. 2D (B+)).

In summary, the behavior of both of these subspecies, living at near extremes of honey bee distribution, differ from other subspecies such as *A.m. ligustica*, *carnica*, and *anatoliaca* (Ben-Shahar et al., 2000; Hadar & Menzel, 2010; Abramson et al., 2015). In these other subspecies similar paradigms result in complete switch from proper response to A+B− to proper response to A−B+, similar to other organisms (Izquierdo et al., 2017).

### The complexity of learning challenge

Using simple conditioning, differences can be observed across drug treatment and control groups (Abramson et al., 2010; Giannoni-Guzmán et al., 2014), but this simple paradigm cannot differentiate age and job-related differences; for instance, across nurse and forager honey bees, or younger and older foraging bees (Ben-Shahar et al., 2000). In these situations, reversal learning paradigms are used to better differentiate the learning abilities that change with age or disease. For example, only during the reversal phase of a reversal learning paradigm could it be shown that dogs and primates exhibit impaired spatial navigation as they age (Lai et al., 1995; Mongillo et al., 2013). In another recent study, reversal learning was necessary to show that an animal model of anorexia nervosa (rat) has impaired cognitive-flexibility, just like the human counterpart (Tchanturia et al., 2011; Allen et al., 2017).

Reversal learning paradigms can probe deeper than its simple conditioning counterpart because it combines two related yet distinct conditioning phases: discrimination and reversal. Thus, we suggest the use of reversal learning paradigms could also be more appropriate when small differences in cognitive performance are expected in other organisms.

### Neural substrates of reversal learning

In studies targeting mechanistic understanding of reversal learning, it is shown that in the first acquisition of rewarded vs non-rewarded stimuli, a type of discrimination learning, vs the second or reversal phase are shown to depend on different neural substrates (Izquierdo et al., 2017, in bees Devaud et al., 2007). The acquisition phase does not require the mushroom body, yet the reversal phase requires the alpha-lobes of the mushroom bodies; as demonstrated by the effects of anesthetics applied directly to this region which only interfere with the reversal phase but not with the acquisition phase (Devaud et al., 2007). Because neuropharmacological studies demonstrate the role of dopamine in reversal learning (Costa et al., 2015), it will be interesting to examine correlates of dopaminergic signaling in the mushroom bodies of *A.m. syriaca* and *A.m. caucasica* bees.

### *A.m. caucasica* vs *A.m. syriaca*

In this study, using the appetitive reversal learning paradigm we demonstrate that *A.m. caucasica* learns new associations, and keeps the previous associations. This is consistent with a highly plastic, generalist foraging behavior. *A.m. syriaca* shows very low plasticity in foraging choice (Çakmak et al., 2010; see Fig. 1), and *A.m. syriaca* does not learn to respond to the reversal CS+ in the appetitive reversal learning paradigm. This is consistent with specialization to one or few resources. Specialization provides for speed of foraging and may reduce exposure to predators during foraging episodes. Foraging modeling (Becher et al., 2014) can help us further dissect the ecological importance of these observed differences.

### Appetitive vs aversive learning

One interpretation of differences across *A.m. syriaca* and *A.m. caucasica* could have been greater learning ability in one vs the other subspecies. However, in that case learning effects would have been expected to be general, such as performance differences in all tasks across the two subspecies. This would be similar to comparing bees treated orally with ethanol and control group bees. For these two groups, both in appetitive and aversive learning tasks the 10% or higher ethanol treatment group performed poorly (Giannoni-Guzmán et al., 2014). However, in a supplemental study (Fig. S2) we demonstrated in an aversive learning paradigm, ESA conditioning, both *A.m. syriaca* and *A.m. caucasica* demonstrated complete reversal of punishment learning. This difference across aversive vs appetitive reversal learning also supports ecological relevance of differences in appetitive reversal learning across subspecies. It is important to note that modality of association cues did not make a difference for the acquisition phase, and demonstrated both subspecies to establish associations for color or odor equally well.

## Conclusion

In this study, we demonstrated a match between the ecology of foraging behavior and learning and memory differences of two honey bee subspecies. As a result we conclude neural substrates of foraging differences may extend beyond modulation of the reward pathway (Giray et al., 2015, Agarwal et al., 2011), and involves learning and memory centers in the brain of the honey bee. In the future, it will be important to compare neurons such as in mushroom bodies and olfactory lobes in the two subspecies, in relation to differences in acquisition and reversal phases in reversal learning (Devaud et al., 2007). Finding the neural substrates linked with the obsessive-like behavior of *A.m. syriaca* will be relevant for other learning contexts and organisms.

## Supplemental Information

10.7717/peerj.5918/supp-1 Supplemental Information 1 Proboscis Extension Response of honeybees from two different subspecies during a Reversal Learning test.

10.7717/peerj.5918/supp-2 Supplemental Information 2 Electric Shock Avoidance data for two honeybee subspecies during a Reversal Learning test. In this data-set 1 means the individual was at the shock side in that particular second, 0 means the individual was not in the shock side.

10.7717/peerj.5918/supp-3 Supplemental Information 3 Proboscis Extension Response data for *A. mellifera* of different ages during a Reversal Learning test. Reversal learning plot in Proboscis Extension Response Conditioning of bees from typical*Apis mellifera*colonies (Ben-Shahar et al., 2000).

10.7717/peerj.5918/supp-4 Supplemental Information 4 Electric Shock Avoidance data for honeybee subspecies *A.m. caucasica* and *A.m. syriaca* during a Reversal Learning test. Comparison of spatial-avoidance learning rate between honey bee subspecies during an ESA assay. Each data point shows the percentage of time (± standard error) bees spent on the shock side during the trial. Both in Acquisition and Reversal phase bees reduce the time spent on shock side over the duration of the training (Repeated measures ANOVA: Acquisition *P*-value < 0.0001, *F*(4,436) = 10.25 and Reversal *P*-value < 0.0001, *F*(4,436) = 6.143. A two-way ANOVA test shows there are no differences between subspecies during Acquisition (*F*(1,109) = 2.315, *P*-value > 0.13) or during Reversal (*F*(1,109) = 0.0065, *P*-value > 0.93).

10.7717/peerj.5918/supp-5 Supplemental Information 5 Supplemental Results for the Electric Shock Avoidance test.

## Additional Information and Declarations

### Data Availability

The following information was supplied regarding data availability: The raw data are available in two Supplemental Files: the PER Data File and the ESA Data File. The PER Data File shows the Proboscis Extension Response conditioning for honey bees in all experimental groups. The ESA Data File shows the Electric Shock Avoidance conditioning data for honey bees in all experimental groups.
