# Supplementary figures and images for: Appetitive reversal learning differences of two honey bee subspecies with different foraging behaviors
Source: PeerJ. 2018 Nov 21;6:e5918. doi: 10.7717/peerj.5918 (PMC6252072; doi:10.7717/peerj.5918)

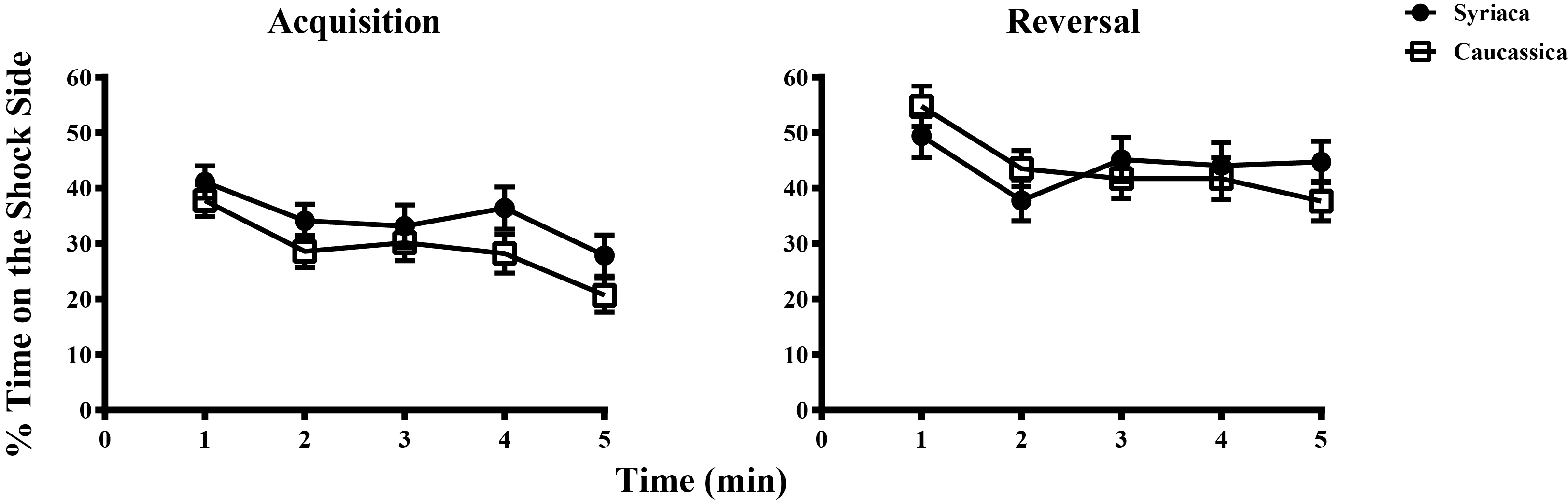

Supplement: Supplemental Information 4 — Comparison of spatial-avoidance learning rate between honey bee subspecies during an ESA assay. Each data point shows the percentage of time (± standard error) bees spent on the shock side during the trial. Both in Acquisition and Reversal phase bees reduce the time spent on shock side over the duration of the training (Repeated measures ANOVA: Acquisition P-value < 0.0001, F(4,436) = 10.25 and Reversal P-value < 0.0001, F(4,436) = 6.143. A two-way ANOVA test shows there are no differences between subspecies during Acquisition (F(1,109) = 2.315, P-value > 0.13) or during Reversal (F(1,109) = 0.0065, P-value > 0.93). [file peerj-06-5918-s004.jpg]
