# Supplementary material for: Appetitive reversal learning differences of two honey bee subspecies with different foraging behaviors
Source: PeerJ. 2018 Nov 21;6:e5918. doi: 10.7717/peerj.5918 (PMC6252072; doi:10.7717/peerj.5918)
Supplement: Supplemental Information 5 [file peerj-06-5918-s005.docx]

**Supplemental Results:**

*Electric shock avoidance (ESA) conditioning*

We used the ESA conditioning assay to determine if the honey bee subspecies *A.m. caucasica* and *A.m. syriaca* have spatial avoidance learning differences. Since individuals can only be on one side of the apparatus at the same time, we only present the data for the CS+. Wilcoxon matched-pairs signed rank test shows there is no significant color preference for *A m. caucasica* between Blue and Yellow for the Initial CS+ (W = -9.00, p = 0.3125; N_1(Blue)_=N_2(Yellow)_=5; Median_1(Blue)_ =19.68; Median _2(Yellow)_= 16.44). Likewise, *A m. syriaca* showed no significant color preference (W = 5.00, p = 0.6250; N_1(Blue)_=N_2(Yellow)_=5; Median_1(Blue)_ =20.63; Median _2(Yellow)_= 21.50). Avoidance learning is indicated by a significant time effect for both Acquisition (F_(4,436)_ = 10.25, ω^2^ = 3.685, p << 0.01; N_1(Syriaca)_ = 56, N_2(Caucasica)_ = 55) and Reversal (F_(4,436)_ = 6.143, ω^2^ = 2.199, p << 0.01; N_1(Syriaca)_ = 56, N_2(Caucasica)_ = 55). Learning performance was lower during reversal phase; a one tailed student t-test shows both *A.m. syriaca* (t_(4)_ = 4.354, p = 0.0121; N_1(Acquisition)_=N_2(Reversal)_=5) and *A.m. caucasica* (t_(4)_ = 14.28, p << 0.01; N_1(Acquisition)_=N_2(Reversal)_=5) spend more time on the shock side during the reversal phase (M = 44.23, SD = 4.186, *syriaca*) (M = 43.88, SD = 6.460, *caucasica*) when compared to the acquisition phase (M = 34.55, SD = 4.828, *syriaca*) (M = 29.10, SD = 6.079, *caucasica*). We found that there are no differences between the learning rates for members of both subspecies during Acquisition (F_(1, 109)_ = 2.315, ω^2^ = 1.186, p = 0.1309; N_1(Syriaca)_ = 56, N_2(Caucasica)_ = 55) or Reversal phases (F_(1, 109)_ = 0.007315, ω^2^ = 0.003891, p = 0.9320; N_1(Syriaca)_ = 56, N_2(Caucasica)_ = 55) (Figure S2).
